# Morbillivirus V Proteins Exhibit Multiple Mechanisms to Block Type 1 and Type 2 Interferon Signalling Pathways

**DOI:** 10.1371/journal.pone.0057063

**Published:** 2013-02-19

**Authors:** Senthil K. Chinnakannan, Sambit K. Nanda, Michael D. Baron

**Affiliations:** The Pirbright Institute, Pirbright, Surrey, United Kingdom; Virginia Polytechnic Institute and State University, United States of America

## Abstract

Morbilliviruses form a closely related group of pathogenic viruses which encode three non-structural proteins V, W and C in their P gene. Previous studies with rinderpest virus (RPV) and measles virus (MeV) have demonstrated that these non-structural proteins play a crucial role in blocking type I (IFNα/β) and type II (IFNγ) interferon action, and various mechanisms have been proposed for these effects. We have directly compared four important morbilliviruses, rinderpest (RPV), measles virus (MeV), peste des petits ruminants virus (PPRV) and canine distemper virus (CDV). These viruses and their V proteins could all block type I IFN action. However, the viruses and their V proteins had varying abilities to block type II IFN action. The ability to block type II IFN-induced gene transcription correlated with co-precipitation of STAT1 with the respective V protein, but there was no correlation between co-precipitation of either STAT1 or STAT2 and the abilities of the V proteins to block type I IFN-induced gene transcription or the creation of the antiviral state. Further study revealed that the V proteins of RPV, MeV, PPRV and CDV could all interfere with phosphorylation of the interferon-receptor-associated kinase Tyk2, and the V protein of highly virulent RPV could also block the phosphorylation of another such kinase, Jak1. Co-precipitation studies showed that morbillivirus V proteins all form a complex containing Tyk2 and Jak1. This study highlights the ability of morbillivirus V proteins to target multiple components of the IFN signalling pathways to control both type I and type II IFN action.

## Introduction

Host innate immune responses to virus infections are initiated by the detection of viral pathogen-associated molecular patterns (PAMPs) (e.g. CpG-DNA, dsRNA, uncapped ssRNA with 5′ triphosphate or specific viral proteins) by cellular pathogen recognition receptors (PRRs) (e.g. Toll-like receptors (TLRs), retinoic acid inducible gene I protein (RIG-I), melanoma differentiation antigen 5 (mda-5) and protein kinase R (PKR)) located on the plasma membrane, in the endosomal compartment or within the cytoplasm of infected cells (reviewed in [1], [2]). This leads to the activation of a complex network of intracellular signalling pathways, which ultimately results in the transcription of host defence genes, particularly pro-inflammatory cytokines including interferons (IFNs) (reviewed in [3]). IFNs are a group of secreted cytokines that induce a virus-resistant state in cells and also play a crucial role in modulating the adaptive immune system. The type I IFNs (primarily IFNα/β), produced in a direct response to virus infection, bind to the common IFNα/β receptor (IFNAR1/IFNAR2c), inducing receptor dimerization, phosphorylation and subsequent phosphorylation of receptor-associated Janus kinases (Jaks), Jak1 and Tyk2. Phosphorylated Jaks then phosphorylate signal transducer and activator of transcription proteins (STATs) STAT1 and STAT2, which are also bound to the receptor subunits. The phosphorylated STAT1 and STAT2 form a heterodimer that associates with a DNA-binding protein called interferon regulatory factor 9 (IRF-9), forming interferon-stimulated gene factor 3 (ISGF3). ISGF3 translocates to the nucleus and binds to a specific sequence called the interferon stimulated response element (ISRE) in the promoter region of interferon stimulated genes (ISGs), activating their transcription which results in the establishment of antiviral state in the cell (reviewed in [3], [4]). The type II IFN (IFNγ), which is secreted by activated T cells and natural killer cells rather than as a direct response to viral infection, mediates its biological action through a different receptor (IFNGR). IFNγ, upon binding to its receptor, induces phosphorylation of the receptor associated Jaks, Jak1 and Jak2. Phosphorylated Jak1/Jak2 then phosphorylate STAT1, which homodimerizes to form gamma activated factor (GAF), which in turn translocates to the nucleus and binds to gamma activated sequence (GAS)-containing promoters and activates transcription of a distinct subset of cellular genes that shape the IFNγ-mediated antiviral response (reviewed in [5]).

Morbilliviruses are a genus in the sub-family *Paramyxovirinae*, order *Mononegavirales*, all of which can cause serious disease in their respective hosts: measles in humans, rinderpest in cattle, peste des petits ruminants in sheep and goats, canine distemper in dogs, mustelids and large felids, phocine distemper in seals, and the cetacean morbillivirus in whales, porpoises and dolphins [6]. Although the most feared of these diseases, rinderpest, has been eradicated from the world, other morbillivirus infections are still a threat to their susceptible host populations [7], [8], [9]. The P gene of morbilliviruses, in addition to the generation of the P protein, also encodes three non-structural proteins; the V and W proteins are produced by co-transcriptional insertion, at a specific editing site about half way along the gene, of additional G residues into a fraction of the mRNAs transcribed from the P gene [10], while the C protein arises from translation of an alternative, completely separate, open reading frame [11]. The P, V and W proteins have a common N-terminal domain which, in the case of the W protein, is essentially the whole of the protein. The P and V proteins have specific C-terminal sequences downstream of the editing site. *In vitro* studies over the last decade with *Measles virus* (MeV), *Canine distemper virus* (CDV) and *Rinderpest virus* (RPV) have shown that the P, V and W proteins have varying abilities to block the IFN signalling pathway [12], [13], [14], [15], [16], [17], [18], [19]. From studies with *Rinderpest virus* (RPV), we have shown that the RPV V protein is a more effective blocker of type I and type II IFN action than the P or W proteins, efficiently inhibiting the phosphorylation of STAT1 and STAT2. Likewise, a recent study has shown that the CDV V protein has similar IFN-antagonistic properties. *In vivo* studies with recombinant MeV and CDV lacking the V or C proteins, in their natural hosts, have demonstrated that V and C are essential mediators of pathogenesis in their host [20], [21], and recombinant MeV in which the V protein was unable to antagonise STAT1 function was found to be attenuated *in vivo*, highlighting the role of the morbillivirus IFN antagonistic proteins in the development of pathogenesis [22].

While it is widely accepted in the literature that the morbillivirus V protein interferes with type I IFN signalling, the effect of this protein on type II IFN signalling has been the subject of debate. Earlier studies [18], [19] reported conflicting observations on MeV V protein's ability to interfere the type II IFN signalling, while more recent studies [13], [14] have shown that the measles V protein could block type II IFN responses depending on the strain of the virus studied and the expression levels of the V proteins in the cell lines tested. We showed that RPV V protein could completely block STAT1/2 phosphorylation induced by either type I or type II IFNs [23]. Very little is known on the mechanistic details underlying the ability of the morbillivirus V proteins to overcome the IFN signalling pathway. Almost all of these *in vitro* studies have found that morbillivirus P, V and W proteins could bind STAT1 and/or STAT2, and it is assumed that this binding governs the ability of morbillivirus V proteins to inhibit either the phosphorylation of STAT1/STAT2 [15], [23], [24], [25] or the nuclear import of the phosphorylated STAT1/STAT2 [18], [26] and hence the block of the IFN signalling pathway. However, there have been only limited study of the abilities of these proteins to block the activation of upstream Jaks or the IFN receptors. There have been two reports, one suggesting that a complex of measles V and C proteins interacts with the type I IFN receptor [24] and another reporting that measles V protein interacts with Jak1 [15]. It is not clear whether the observed blockade of IFN signalling is due to interference in the activation step at the receptor (e.g. activation of Jak1/Tyk2), which in turn could result in a block of the phosphorylation and nuclear accumulation of STAT1/STAT2, or directly at the level of STATs, or both. Detailed studies to understand the molecular mechanisms underlying the abilities of these proteins to block IFN action would be useful for the development of mutant viruses specifically lacking IFN-antagonistic properties and also in the development of successful anti-viral intervention strategies. Here we compare the V proteins from four different morbilliviruses and show that all the morbillivirus V proteins can block type I IFN action, although their effectiveness at preventing the induction of the anti-viral state varied, and depended to some extent on the kind of assay employed. On the other hand, we found that morbillivirus V proteins can show very different abilities to block type II IFN action and that the viruses appear to use different mechanisms in blocking the type I and type II IFN signalling pathways. Our study suggests that the observed variation in the abilities of different morbillivirus V proteins to block type I and/or type II IFN action might be related to their varying ability to target distinct components of the IFN signalling pathways.

## Results

### Different morbilliviruses show different abilities to block type I and type II IFN-induced phosphorylation and nuclear translocation of STAT1/STAT2

In previous studies [23] we have shown that RPV blocks the IFN-induced phosphorylation of STAT1 and STAT2. In order to understand the mechanisms underlying the ability of RPV and other morbilliviruses to block IFN action, we carried out comparative studies with four important morbilliviruses, RPV, MeV, PPRV and CDV. We were unable to achieve the high levels of infection (>90%) required for looking at total STAT1/STAT2 phosphorylation in infected cells with any virus except RPV-Sa. We therefore performed immunofluorescence studies to analyze STAT1 and STAT2 activation in cells infected with wild-type strains of the four morbilliviruses. In addition to the RPV-Sa strain, we used the Dublin 2000 strain of MeV [27] (MeV-Dublin), the Turkey/2000 strain of *Peste des petits ruminants virus* (PPRV) (PPRV-Tu) and the 5804p strain of CDV [28] (CDV-5804p). A549 cells were infected with these viruses and, 20 hours post-infection, cells were stimulated with either IFNα or IFNγ for 30 minutes, fixed, stained and observed for the presence or absence of phosphorylated STAT1 or nuclear STAT2 (note that we were unable to get satisfactory results with antibody to phosphorylated STAT2, so took STAT2 accumulation in the nucleus as a measure of the phosphorylation of this protein, and prevention of STAT2 accumulation as an indication of a block of STAT2 phosphorylation, although it remains possible that STAT2 could be being phosphorylated in some of our studies but being prevented from entering the nucleus). A hundred randomly selected infected cells were analyzed for each virus to obtain a quantitative measure on the ability of these viruses to block the IFN-induced activation of STAT1 and STAT2 [23].

**Figure 1:**
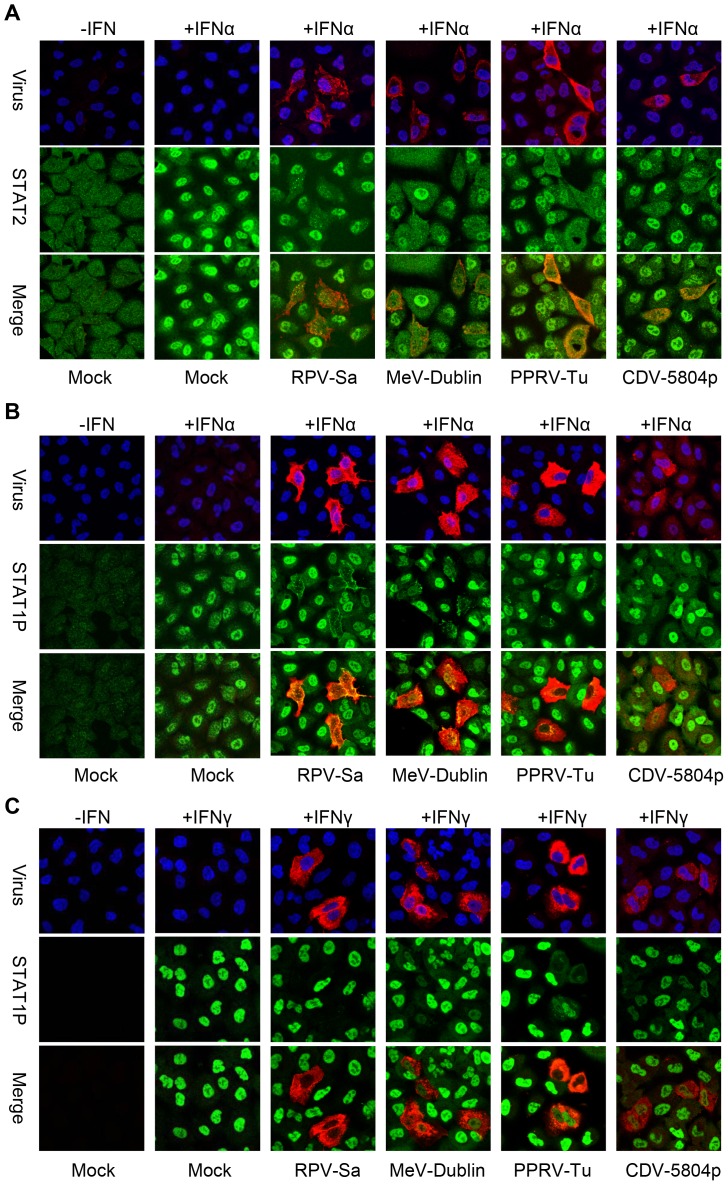
All four morbilliviruses efficiently blocks IFNα-induced STAT2 phosphorylation, but show varied abilities to block IFNα and INF-γ induced STAT1 phosphorylation. A549 cells were mock infected or infected with the indicated morbillivirus as in “Material and methods”. Eighteen hours post-infection cells were mock treated or treated with 1000 IU/ml of IFNα (a), (b) or 1000 IU/ml of IFNγ (c) for 30 minutes, fixed with PFA and methanol and stained with (a) rabbit anti-STAT2 plus goat anti-PPRV or (b, c) mouse anti-phospho-STAT1 (STAT1P) plus rabbit anti-RPV. Representative confocal images from three independent experiments are shown.

Interestingly, all the four different morbilliviruses studied showed 100% efficiency in blocking IFNα induced activation (nuclear accumulation) of STAT2 (Fig. 1a); however, they showed varying abilities to block IFNα or IFNγ induced STAT1 phosphorylation (Fig. 1b, 1c). In general, all the four morbilliviruses showed a high to intermediate efficiency in blocking the IFNα induced STAT1 phosphorylation, with RPV-Sa and PPRV-Tu being clearly more effective than the other two viruses. Quantitatively, RPV-Sa and PPRV-Tu showed a 95% block (Fig. 1b); the small percentage (5%) of RPV-Sa and PPRV-Tu infected cells in which phosphorylated STAT1 could be seen in the nucleus appeared to be in early stages of infection, as judged by lower levels of viral proteins in such cells, and this could have resulted in their inability to completely block the IFNα signal. Approximately 60% of cells infected with MeV-Dublin or CDV-5804p showed a complete block in IFNα induced STAT1 phosphorylation (61% and 57% respectively) (Fig. 1b). Further infected cells showed a reduction in the apparent amount of phosphorylated STAT1 compared to neighbouring uninfected cells, although this was not quantifiable.

The morbilliviruses studied showed a much wider variation in their ability to block the IFNγ induced phosphorylation of STAT1 (Fig. 1c). All four viruses showed some activity, but only RPV-Sa infected cells showed a complete block, with no STAT1P detected in infected cells. In contrast, many cells infected with MeV-Dublin, PPRV-Tu or CDV-5804p, even cells showing high levels of infection, showed only a partial block, with some STAT1P seen in the nucleus. Quantitating the viruses' effects based on the presence or absence of STAT1P in the virus infected cells, RPV-Sa, MeV-Dublin, PPRV-Tu and CDV-5804p infected cells showed 90%, 16%, 55% and 11% block in IFNγ-induced STAT1 phosphorylation, respectively. All the viruses, therefore, appear to be able to block IFNγ signalling, though RPV-Sa appears to be quantitatively much more effective at this than the other viruses (the quantitative data from these and other immunofluorescence studies are summarised in Table S1). Importantly, there was no correlation between the ability of the viruses to block phosphorylation of STAT1 induced by IFNα or IFNγ, suggesting that there may be different mechanisms underlying these two effects.

### The V proteins of RPV, MeV, PPRV and CDV can all block type I IFN action but show varied abilities to block type II IFN action

We then compared the V proteins from these viruses to analyze the similarities and differences between the different morbillivirus V proteins in their abilities to block IFN action. For this purpose we generated plasmid constructs encoding V5 epitope-tagged V proteins from MeV-Dublin, PPRV-Tu, and CDV-5804p for comparison with the V protein of RPV-Sa. In addition, for some of the studies we also included plasmids encoding the V5 epitope-tagged V proteins of vaccine strains of different morbilliviruses: MeV Edmonston strain (MeV-Edm), CDV Onderstepoort strain (CDV-Ond), RPV-RBOK. All the constructs were engineered to prevent C protein expression from the overlapping reading frame. These proteins were then compared for their abilities to block type I and type II IFN action using various assays: reporter gene assays for IFN-induced transcription, immunofluorescence assays for IFN-induced phosphorylation of STAT1/2, a functional assay of IFN-induced establishment of the antiviral state and direct measurement of binding of STAT1 and STAT2 by the different V proteins using co-immunoprecipitation.

**Figure 2:**
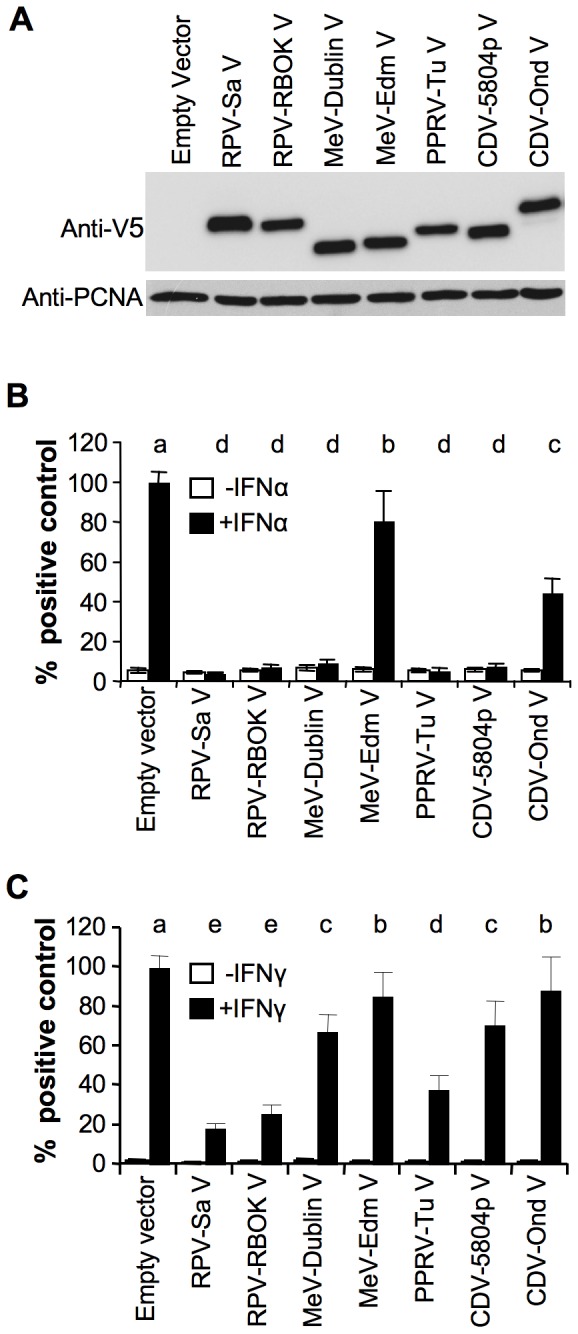
Abilities of morbillivirus V proteins to block type I and type II IFN induced gene transcription. (a) Vero human-SLAM cells were transfected with 0.5 µg of empty vector or plasmid driving the expression of the indicated protein. Twenty-four hours post transfection, cells were lysed in SDS-PAGE sample buffer and the expressed proteins were detected by Western blot using mouse anti-V5 antibody. PCNA levels served as loading control. (b), (c) Vero human-SLAM cells were transfected with 0.5 µg each of (b) pGL3-MX1P-luc or (c) pGAS-luc, together with pJATLacZ and either empty vector or plasmid driving the expression of the indicated protein. Twenty-four hours post-transfection, cells treated with either (b) 1000 IU/ml of IFNα or (c) 1000 IU/ml of IFNγ or left untreated, lysed and assayed for luciferase and β-galactosidase activities as described in Methods. Letters above the bars for the IFN-treated samples indicate the results of statistical analysis: results which were not statistically different from one other have the same letter.

All the morbillivirus V proteins expressed at similar levels from transfected plasmids (Fig. 2a). Although all the morbillivirus V proteins used in this study are composed of 300 amino acids, with the exception of PPRV-Tu V (299 amino acids), they all appeared to have different molecular weights by SDS-PAGE, an observation previously made for V proteins of different MeV isolates [13], [14]. The source of this variation is not clear, but is probably due to the relatively high negative charge of the P/V shared domain, which leads to aberrant migration of these proteins (P, V and W) on SDS-PAGE gels (e.g. [29], [30], [31]). Reporter assays for type I IFN-induced gene transcription with the pGL3-MX1P-luc reporter plasmid showed that, with the exception of the V proteins from the vaccine strains of MeV (MeV-Edm V) and CDV (CDV-Ond V), all the morbillivirus V proteins studied were effective blockers of type I IFN action (Fig. 2b). MeV-Edm V showed only a weak inhibition of luciferase induction (∼20%), though this was statistically significant (p<0.01), while the CDV-Ond V protein showed an intermediate effect (∼50%). It is known from published data that the V protein from MeV-Edm is disabled in its IFN antagonistic properties [17], so the behaviour of the MeV-Edm V protein in this assay is not surprising, but it appears that the V protein of the vaccine strain of CDV (CDV-Ond V) is also relatively poor in its ability to block IFNα action. However, the vaccine strain of RPV (RPV-RBOK V) retained the ability to block IFNα induced transcription. Interestingly, similar to the observations from the immunofluorescence study with the virulent strains of the four different morbilliviruses, the morbillivirus V proteins also showed wide variation in their abilities to block type II IFN action (Fig. 2c). The RPV V proteins (both RPV-Sa V and RPV-RBOK V) showed the strongest inhibition, followed by, in decreasing order of activity, the PPRV-Tu V protein, then the MeV-Dublin V and CDV-5804p V proteins (not significantly different from each other), and lastly the MeV-Edm V and CDV-Ond V proteins (also not significantly different from each other).

**Figure 3:**
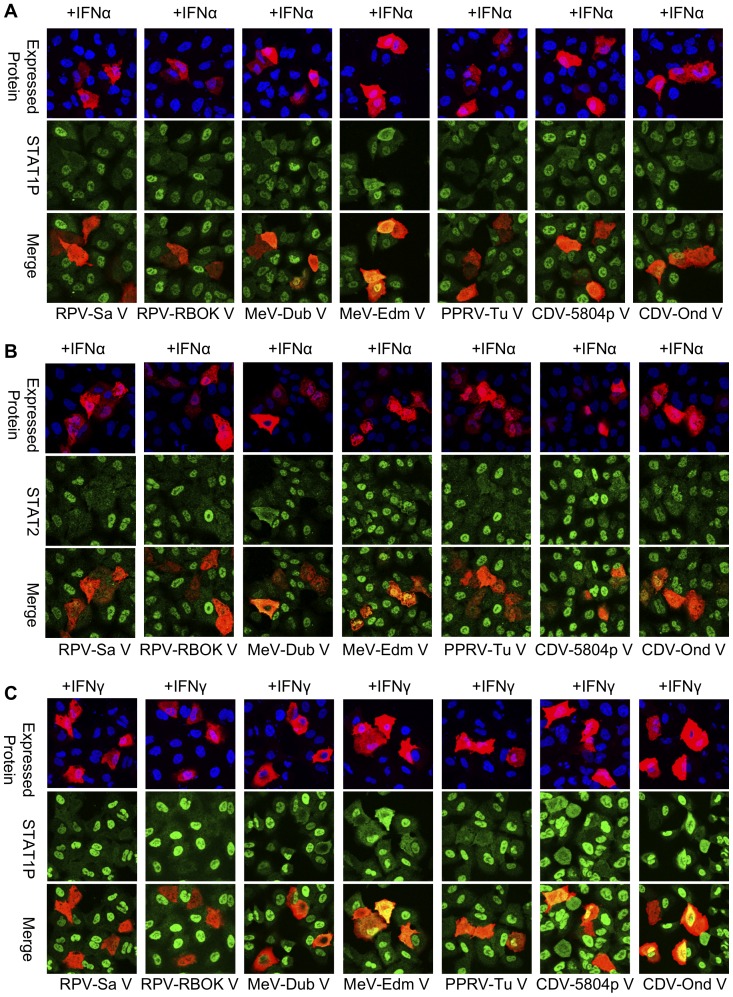
Morbillivirus V proteins show better ability to block IFNα-induced STAT1 and STAT2 phosphorylation than INFγ-induced STAT1 phosphorylation. A549 cells were transfected with 1 µg of empty vector or plasmid driving the expression of the indicated protein. Twenty hours post-transfection, cells were mock treated or treated with (a),(b) 1000 IU/ml of IFNα or (c) 1000 IU/ml of IFNγ for 30 minutes, fixed with PFA followed by methanol and stained with monoclonal anti-V5 tag and (a), (c) anti-phospho-STAT1 (STAT1P) or (b) anti-STAT2 as in “Material and methods”.. Representative confocal images from three independent experiments are shown.

When the morbillivirus V proteins were compared for their abilities to block IFN-induced STAT1/STAT2 phosphorylation, we found that all the morbillivirus V proteins studied, except the V proteins from MeV-Edm and CDV-Ond, were efficient blockers of type I IFN induced STAT1/STAT2 phosphorylation. Quantitatively, on counting a hundred randomly chosen cells expressing the V protein of interest, RPV-RBOK V showed 100% and 94% of cells with a complete block in IFNα-induced phosphorylation of STAT1 and STAT2 respectively (essentially the same as RPV-Sa V, as shown in Fig. 2a, 2c). The corresponding values for the other V proteins were: MeV-Dublin, 87% and 94%; PPRV-Tu, 94% and 94%; CDV-5804p, 90% and 93%; CDV-Ond, 55% and 48%; MeV-Edm, 43% and 18% (Fig. 3a, 3b). However, similar to the observations from the reporter assays, the V proteins showed varying abilities to block type II IFN-induced STAT1 phosphorylation. RPV-RBOK V, as with RPV-Sa V, blocked STAT1 phosphorylation in all expressing cells, with the corresponding values for the other V proteins being PPRV-Tu, 72%; CDV-5804p, 53%; MeV-Dublin, 51%; CDV-Ond, 40% and MeV-Edm, 37% (Fig. 3c). There was thus good correlation between the relative ability of the V proteins to inhibit IFNγ-induced STAT1 phosphorylation and the ability to block induction of transcription from the IFNγ-sensitive GAS promoter.

**Figure 4:**
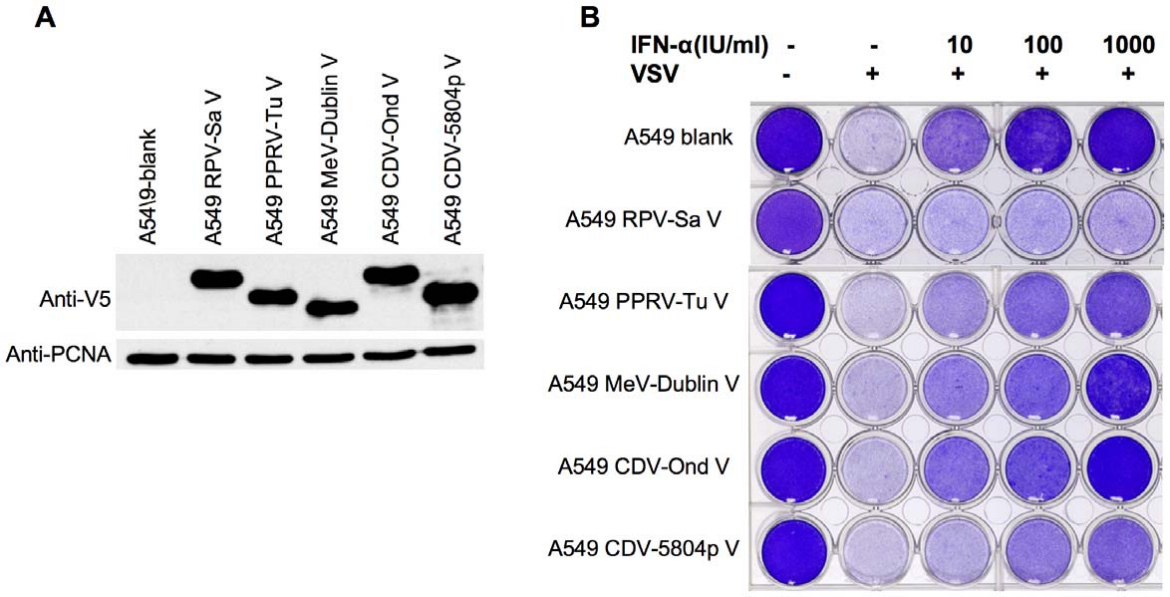
All four morbillivirus V proteins have the ability to block the induction of the type I IFN-induced antiviral state. (a) Demonstration of expression of different morbillivirus V proteins in the stable cell lines used for the VSV CPE functional assays. A549-blank and stable cell lines expressing the indicated proteins were plated in 24-well plates. Next day, cells were lysed in SDS-PAGE sample buffer and the expressed proteins were detected in Western blots using mouse anti-V5 antibody. PCNA levels served as loading control. (b) VSV CPE assay for the type I IFN-induced antiviral state. Stable cell lines expressing the indicated proteins were plated in 24-well plates. Next day, cells were mock treated or treated with 10, 100, 1000 IU/ml of IFNα for 24 hours. The cells were then challenged with VSV at a MOI of 0.1 for 24 hours (or left untreated), fixed and stained with crystal violet. Shown are results from one of three independent experiments.

In order to test the actions of the V proteins in a possibly more biologically relevant assay, we also generated cell lines expressing these proteins and tested their abilities to block the induction of the antiviral state mediated by type I IFN. This was done by treating the cells with different concentrations of IFNα and then challenging them with vesicular stomatitis virus (VSV). The development of the antiviral state in the cells is shown by the prevention of VSV-induced cytopathic effect and cell death. A cell line stably transduced with the empty vector (A549-blank) was generated to act as a negative control. We made cell lines expressing the V proteins of each of the wild type viruses as well as one expressing CDV-Ond V, and analyzed their abilities to block the development of the IFNα-induced antiviral state (given the known defects in the V protein of MeV-Edm [17], confirmed in our own studies above, we did not make a cell line expressing this protein). Fig. 4a shows the relative expression levels of different V proteins in these cell lines. The results of the VSV c.p.e reduction assay showed that all five morbillivirus V proteins tested could block the induction of the type I IFN mediated antiviral state at lower amounts of IFNα stimulation (10 or 100 IU/ml) (Fig. 4b, column 3, 4). However, differences between the V proteins became apparent at the highest amounts of IFNα stimulation (1000 IU/ml); the CDV-Ond V protein showed no apparent effect at this concentration of IFN and the other four morbillivirus V proteins tested (RPV-Sa V, MeV-Dublin V, PPRV-Tu V and CDV-5804p V) interfered with the induction of the antiviral state with differing efficiencies (Fig. 4b, column 5), with MeV-Dublin V having the lowest efficiency. The order in their abilities to block the induction of type I IFN mediated antiviral state can be represented as RPV-Sa V>CDV-5804p V≥PPRV-Tu V>MeV-Dublin V>CDV-Ond V.

### Morbillivirus V proteins show varied abilities to bind STAT1/STAT2

**Figure 5:**
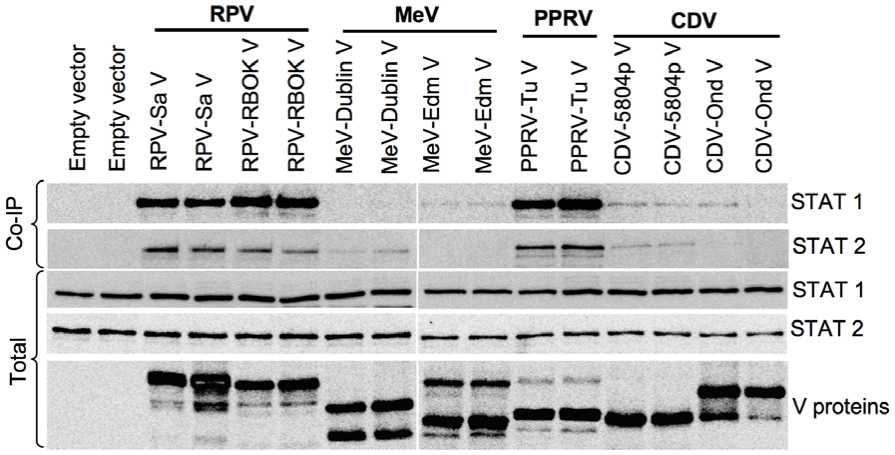
Morbillivirus V proteins show varied abilities to co-precipitate STAT1 and STAT2. Vero human-SLAM cells were transfected in duplicate wells with 1 µg of empty vector or plasmid driving the expression of the indicated protein. Forty-eight hours post-transfection, one member of each pair was lysed with lysis buffer at pH 7.5 and second with lysis buffer at pH 8.0. The lysates were immunoextracted with mouse anti-V5 antibody as in “Materials and methods”. The immunoprecipitates (Co-IP) and a fraction of total cell lysate (1/10th) were analyzed in Western blots for the presence of STAT1 or STAT2 using rabbit anti-STAT1 and rabbit anti-STAT2 antibodies respectively. The blots from total cell lysates were also probed with mouse anti-V5 antibody for analyzing the expression levels of the expected V proteins. The primary antibodies were detected with peroxidase-labelled anti-mouse or anti-rabbit IgG antibody.

To try to understand the mechanism underlying the ability of different morbilliviruses to block type I and type II IFN action, we performed STAT1 and STAT2 co-immunoprecipitation studies with these proteins. We found that the different morbillivirus V proteins in our study co-precipitated STAT1 and STAT2 with different efficiencies, suggesting that they may have different affinities for the host cell proteins (Fig. 5). The V proteins of RPV (RPV-Sa V and RPV-RBOK V) clearly co-precipitated both STAT1 and STAT2, and that of PPRV (PPRV-Tu V) was equally effective at co-precipitating both STATs. In contrast, MeV-V proteins (MeV-Dublin V and MeV-Edm V) and CDV-V proteins (CDV-5804pV and CDV-Ond V) showed much lower co-precipitation of STAT1 and STAT2, and in some cases no apparent binding, despite good expression of all the relevant V proteins. Notably, MeV-Dublin V precipitated only minute amounts of STAT1, barely detectable on Western blots, while the MeV-Edm and CDV-Ond V proteins failed to precipitate detectable STAT2. Previous studies have shown MeV V protein binding both STAT1 and STAT2 [12], [14], [15]. Co-precipitation studies with MeV V protein in the above mentioned work were performed using a cell lysis buffer at pH 8.0; in our studies, we use a lysis buffer at pH 7.5 (which is nearer the physiological pH), though otherwise very similar in composition. To investigate whether the difference in the pH of the lysis buffer in our experiments and those published by others contributed to the difference in the ability to co-precipitate STAT1 and STAT2 by MeV V and CDV V proteins, we performed STAT1 and STAT2 co-precipitation experiments in duplicate, one using lysis buffer at pH 7.5 and another using lysis buffer at pH 8.0. As can be seen in Fig. 5, the difference in pH had very little effect on STAT1/2 co-precipitation, except to reduce the co-precipitation of STAT1 with CDV-Ond V at pH 8.0.

We did not observe any correlation in the amounts of STAT1 and STAT2 co-precipitated with the different V proteins. Although STAT1 and STAT2 were detected with different antibodies, and therefore we cannot determine the actual ratio of STAT1 to STAT2 in any co-precipitate, we could use the light signal emitted from the Western blots to compare the ratio between V proteins; we found signal ratios (STAT1∶STAT2) from STAT1 and STAT2 associated with the different V proteins varying from 7.8 (RPV RBOK) to 0.3 (MeV Dub). This suggests that we are not looking simply at varying co-precipitation of a specific cellular complex containing both STAT1 and STAT2. These results are more likely to result from different binding sites for the two STATs on the V protein, as has previously been suggested [12], [14], [32], [33], [34]. We also observed that, while all the wild type V proteins appear to bind some STAT2 (with RPV and PPRV V proteins being 2-3-fold more effective), there is a much bigger difference in the apparent ability to bind STAT1, with RPV and PPRV V proteins co-precipitating many times more STAT1 than CDV-5804p V, which in turn brought down more than MeV-Dub V.

### RPV infection blocks IFNα induced phosphorylation Jak1/Tyk2

**Figure 6:**
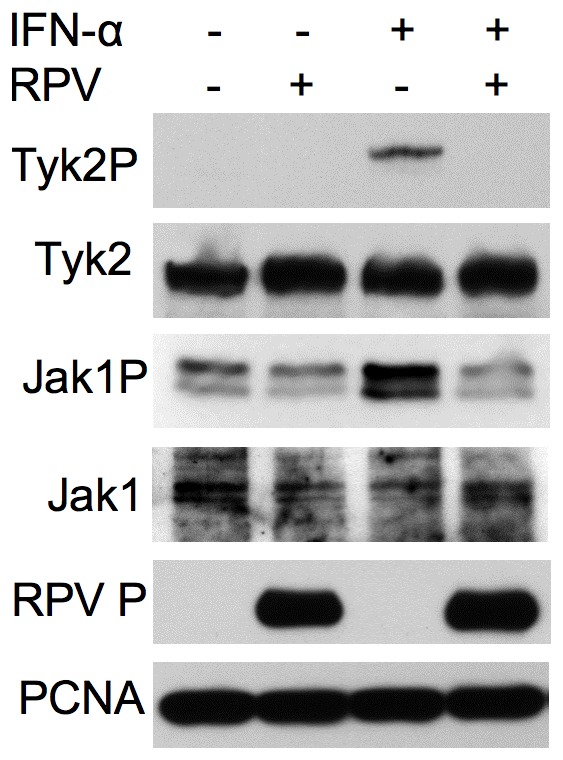
RPV-Sa infection blocks IFNα-induced phosphorylation of Jak1/Tyk2. A549 cells were infected with RPV-Sa at a MOI of 5 or left uninfected. Eighteen hours post-infection cells were mock treated or treated with 1000 IU/ml of IFNα for 15 minutes, lysed in SDS-PAGE sample buffer containing protease and phosphatase inhibitors and the levels of phosphorylated Jak1 and Tyk2 were analyzed in Western blots using rabbit anti-Jak1P and rabbit anti-Tyk2P antibodies respectively. The blots were also probed with rabbit anti-Jak1, rabbit anti-Tyk2 and rabbit anti-RPV P protein (MB18) to analyze the total Jak1 and Tyk2 and to confirm RPV infection. The primary antibodies were detected with peroxidase-labelled anti-mouse or anti-rabbit IgG antibody. PCNA levels served as loading control.

The results from these studies showed that all the morbillivirus V proteins studied, to a certain extent, can inhibit type II IFN action, and the ability to block type II IFN action in the reporter gene assays and as assessed by inhibition of STAT1 phosphorylation approximately correlated with the ability to co-precipitate STAT1. In contrast, there was no correlation between the abilities of the V proteins to co-precipitate STAT1/STAT2 and their abilities to block type I IFN action. This suggested that the V proteins might have an additional mechanism to block the type I IFN signalling pathway. Since the different morbilliviruses and their V proteins were all effective blockers of STAT2 phosphorylation, one possibility was that the V proteins block the activation (phosphorylation) of the receptor associated kinases Jak1 and/or Tyk2, since this would lead to inhibition of STAT2 phosphorylation irrespective of affinity for STAT2 itself. To further investigate this, we studied the ability of RPV-Sa to block the IFNα induced phosphorylation of Jak1/Tyk2 in A549 cells. When compared to uninfected cells treated with IFNα, RPV-Sa infected cells treated with IFNα showed a complete block in the phosphorylation of Jak1 and Tyk2 (Fig. 6). Unfortunately, a similar study with the other morbilliviruses in our study was not possible as we were unable to get high levels of infection of A549 cells with these viruses, even when using high MOI, and the A549 cells were the only cells of those we tried that had sufficient native Jak1 and Tyk2 for their phosphorylated forms to be detected by Western blot. However, the results with RPV showed that the virus can directly or indirectly block the initial steps of the type I IFN response pathway, and it was possible that this was the mechanism behind the effect of the V proteins on STAT2 phosphorylation.

### The V proteins of RPV, MeV, PPRV and CDV inhibit phosphorylation of Tyk2, however only the RPV-Sa V protein inhibits phosphorylation of Jak1

We therefore studied the ability of different morbillivirus V proteins to block Jak1 and Tyk2 phosphorylation. To do this we utilised the observation that even slight over expression of Jak1 or Tyk2 proteins leads to their phosphorylation; this has previously been used to study the activity of the Marbug virus VP40 protein [35], where it was shown that co-expression of VP40 along with Jak1 or Tyk2 resulted in a block of this phosphorylation. We created plasmid constructs encoding FLAG-tagged Jak1 or Tyk2 and transfected these into cells along with the plasmids encoding the morbillivirus V proteins of interest. The amounts of the Jak1 and Tyk2 expression plasmids transfected were the smallest amount that gave clear phosphorylated kinase protein in our Western blots (note that we consistently detected two phospho-Tyk2 bands in Vero cells with the polyclonal antibody used). In addition to a negative control consisting of empty expression vector, we included a second negative control consisting of a plasmid encoding a protein previously found in our hands to have no effect on type I and type II IFN action, the nucleocapsid (N) protein of Ganjam virus [36], to control for any effect of simply co-expressing a relatively large amount of another protein.

**Figure 7:**
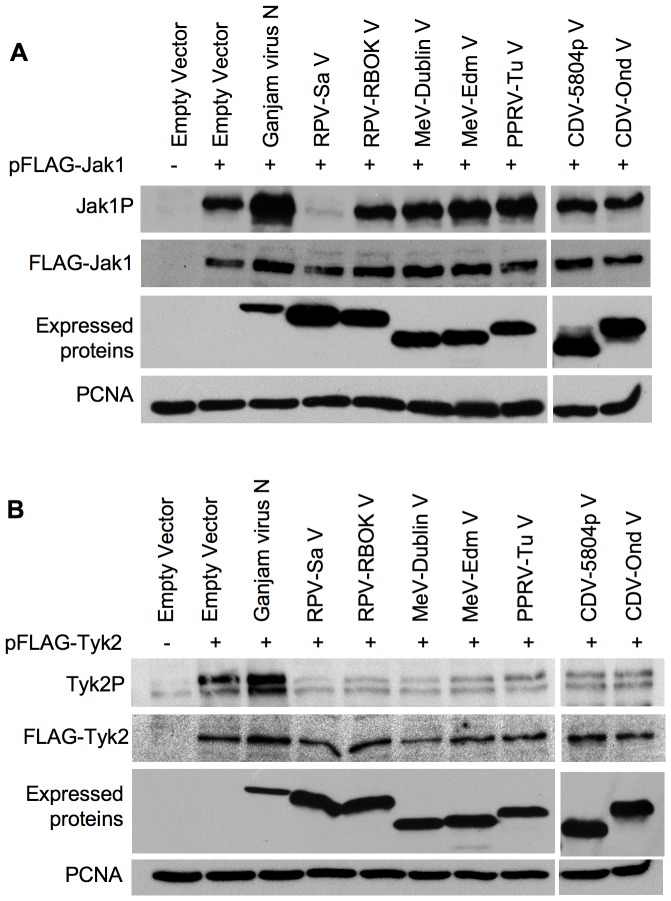
Inhibition of Jak1 and Tyk2 over-expression-induced phosphorylation By morbillivirus V proteins. (a) Vero human-SLAM cells were co-transfected with empty vector or plasmid encoding the indicated protein along with 300 ng pFLAG-Jak1. (b) Vero dog-SLAM cells were co-transfected with empty vector or plasmid encoding the indicated protein along with 400 ng pFLAG-Tyk2. Twenty-four hours post-transfection, the cells were lysed in SDS-PAGE sample buffer containing protease and phosphatase inhibitors as described in “Materials and methods” and the levels of phosphorylated Jak1 and phosphorylated Tyk2 were analyzed in Western blots using rabbit anti-Jak1P and rabbit anti-Tyk2P antibodies respectively. The blots were also probed with mouse anti-FLAG and mouse anti-V5 antibodies to analyze the expression levels of FLAG-Jak1 or FLAG-Tyk2 and the expected morbillivirus V proteins. The primary antibodies were detected with peroxidase-labelled anti-mouse or anti-rabbit IgG antibody. PCNA levels served as loading control.

Interestingly, except for the V protein from the virulent strain of rinderpest virus (RPV-Sa), none of the morbillivirus V proteins studied (RPV-RBOK, MeV-Dublin, MeV-Edm, PPRV-Tu, CDV-5804p and CDV-Ond) showed any effect on the phosphorylation of Jak1 (Fig. 7a). In contrast, all the morbillivirus V proteins could effectively block the phosphorylation of Tyk2 (Fig. 7b). Co-transfection of cells with plasmid encoding FLAG-Tyk2 and either empty vector or plasmid encoding the Ganjam virus N protein showed a clear phosphorylation of Tyk2, whereas co-transfection of cells with the FLAG-Tyk2 plasmid along with plasmids encoding the different morbillivirus V proteins showed a strong inhibition of Tyk2 phosphorylation. There were approximately similar amounts of FLAG-Jak1 or FLAG-Tyk2 expression in all the samples, showing that there was no suppression of Jak1/Tyk2 expression by any of the V proteins. These data suggest that, although all the morbillivirus V proteins have the ability to block Tyk2 activity/phosphorylation, the V protein of the highly virulent RPV-Sa has an additional ability allowing it to also inhibit Jak1 activity/phosphorylation. These data suggest that the observed block of Jak1/Tyk2 phosphorylation in RPV-Sa-infected cells is a property of the V protein.

**Figure 8:**
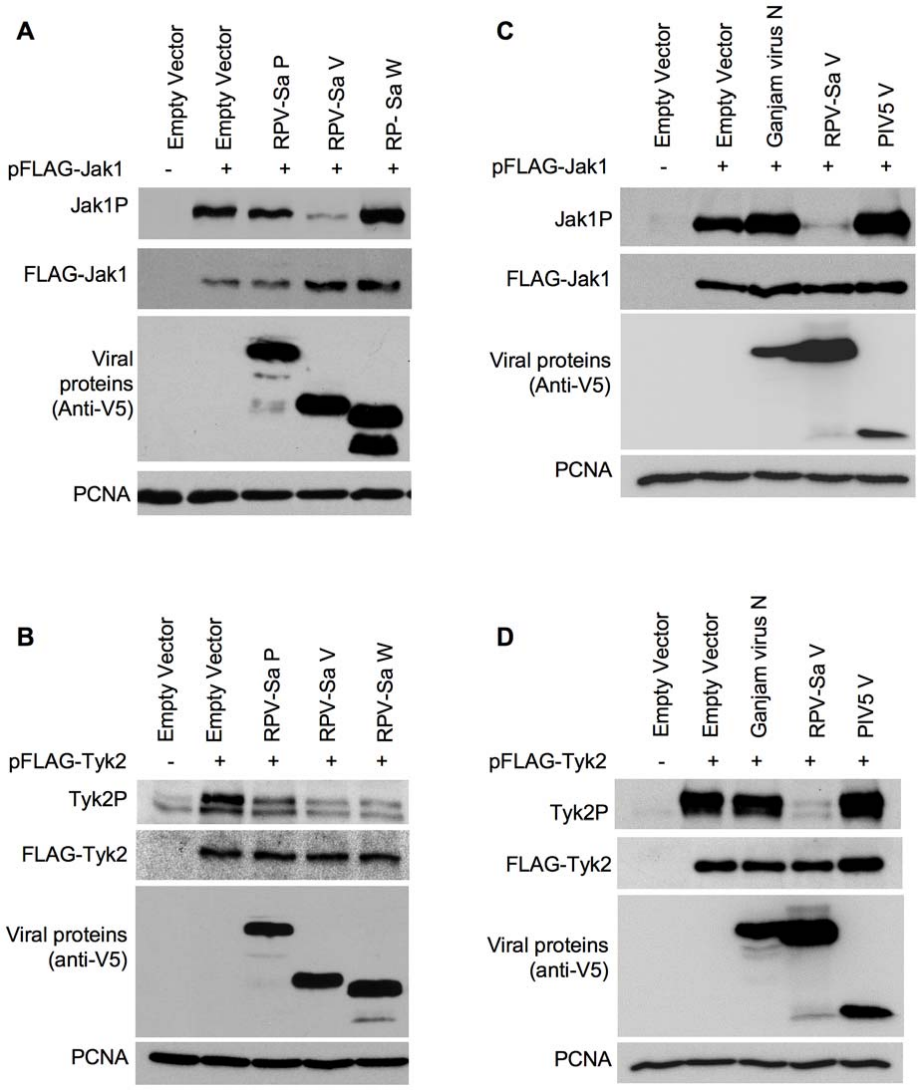
RPV-Sa V but not RPV-Sa P or W, nor the PIV5 V, blocks Jak1 and Tyk2 phosphorylation. (a), (c) Vero human-SLAM cells were co-transfected with pFLAGJak1 and plasmids encoding the indicated viral proteins, and Jak1 phosphorylation determined, as described for Fig. 8 (a). (b), (d) Vero dog-SLAM cells were co-transfected with pFLAGTyk2 and plasmids encoding the indicated viral proteins, and Tyk2 phosphorylation determined, as described for Fig. 8 (b).

To see which part of the V protein was involved in these effects on Jak1 and Tyk2, we also analyzed the ability of the RPV-Sa P and W proteins to block Jak1/Tyk2 phosphorylation. The P and W proteins showed no significant effect on Jak1 phosphorylation (Fig. 8a), however, these proteins did block Tyk2 phosphorylation, and the effect of P was slightly less than that of W (Fig. 8b), suggesting that the mechanisms behind the blockade of Tyk2 and Jak1 are different, since the P/V/W domain can block Tyk2 phosphorylation, but an intact V is required for the effect on Jak1. As with the V proteins, expression of RPV-Sa P or W did not have any effect on FLAG-Jak1 or FLAG-Tyk2 expression.

We also looked to see if the effects of the morbillivirus V proteins on Jak1 and/or Tyk2 phosphorylation are specific for that genus or if it is found in the V proteins of other paramyxoviruses. We performed a preliminary study with the V protein of parainfluenza virus type 5 (PIV5), a member of the genus *Rubulavirus*. This V protein is known to use a different mechanism to inhibit IFN action, targeting STAT1 protein for proteasomal degradation [37], [38]. The PIV5-V protein had no effect on the phosphorylation of overexpressed Jak1/Tyk2 (Fig. 8c and 8d), suggesting that the ability of morbillivirus V proteins to block Jak1 and/or Tyk2 phosphorylation is not common to all the paramyxovirus V proteins, unlike the ability to bind mda5 protein [39]. It will be interesting to look at the V proteins of viruses of other paramyxovirus genera, the avulaviruses, henipaviruses and respiroviruses.

### Morbillivirus V proteins form complexes with Jak1 and Tyk2

**Figure 9:**
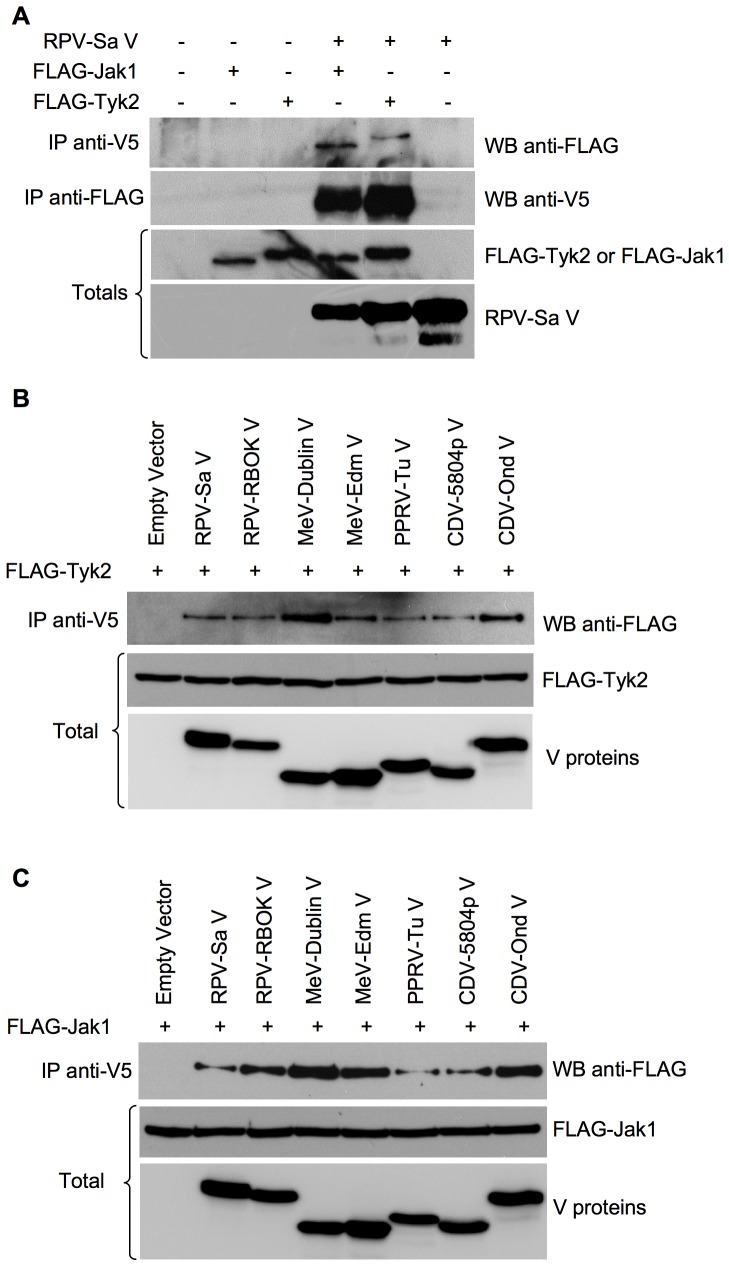
All morbillivirus V proteins co-precipitate Jak1 and Tyk2. (a) Vero human-SLAM cells (b), (c) HEK 293FT cells were co-transfected with 1 µg of empty vector or plasmids encoding the indicated proteins along with 2 µg pFLAG-Jak1 or pFLAG-Tyk2. Forty-eight hours post-transfection, cells were lysed with NP-40 lysis buffer (pH 7.5) and the lysates were immunoextracted with mouse anti-V5 or mouse anti-FLAG antibodies as described in “Materials and methods”. The immunoprecipitates and the total cell lysate (1/10) were analyzed in Western blots for the presence of FLAG-Jak1 or FLAG-Tyk2 and the expected V proteins. The primary antibodies were detected with peroxidase-labelled anti-mouse IgG antibody.

Since we observed that morbillivirus V proteins can interfere with the phosphorylation of Jak1 and/or Tyk2, we performed co-immunoprecipitation experiments to look for a physical interaction of the V proteins with either Jak1 or Tyk2. We first analyzed if the RPV-Sa V could co-precipitate Jak1 or Tyk2 and vice versa. For this purpose the V5-tagged RPV-Sa V was co-expressed with either FLAG-Jak1 or FLAG-Tyk2 and immunoprecipitated with mouse anit-V5 or mouse anti-FLAG antibodies. The results showed that the RPV-Sa V protein could co-precipitate both Jak1 and Tyk2, and further that the Jak1 and Tyk2 proteins could also co-precipitate the RPV-Sa V protein, when these proteins are co-expressed (Fig. 9a). We then performed similar experiments to compare the Jak1/Tyk2 co-precipitation abilities of the other morbillivirus V proteins. All the morbillivirus V proteins co-precipitated Tyk2 (Fig. 9b). Interestingly, all the morbillivirus V proteins also co-precipitated Jak1 (Fig. 9c). It is not yet clear whether the V proteins have a direct or indirect interaction with Jak1/Tyk2 proteins, and it is possible that the morbillivirus V proteins form a complex interaction at the IFN receptor involving Jak1/Tyk2, STAT1/STAT2 and possibly other proteins as shown by Yokota et al., 2003 and hence, when the V protein is pulled down, Jak1 or Tyk2 is also being co-precipitated in the complex. The ready precipitation of V with Jak1 or Tyk2, and vice versa, when only one of Jak1 or Tyk2 is being overexpressed, does suggest that the interaction may be direct, but further studies are required to understand the implications of these interactions on the activation of the IFN signalling pathway.

## Discussion

Viruses in the sub-family *Paramyxovirinae* have adopted various strategies to block the IFN signalling pathways. While most of the members of the genus *Rubulavirus* and *Avulavirus* target one or more STAT proteins for degradation [37], [38], [40], members of the genus *Henipavirus*, *Morbillivirus and Respirovirus* use different strategies; they do not induce STAT degradation, but rather affect the phosphorylation and/or nuclear accumulation of the phosphorylated STAT1 and STAT2. In respiroviruses, the non-structural C protein has been implicated in the block of IFN signalling [41], [42], [43], whereas in the henipaviruses [30], [44], [45] and morbilliviruses [18], [19], [23], [26], it is the V protein, characterised by the presence of a highly-conserved motif containing seven cysteine residues in their V-specific domains, that mediates IFN evasion activities (reviewed in [46], [47], [48], [49]).

As outlined in the introduction, there is some variation in the results of studies on the activities of morbillivirus V proteins, both between different viruses and between laboratories working on the same virus (but using different strains and assays). To improve our understanding of the mechanism(s) involved in the ability of morbillivirus V protein to block IFN action, we compared four different morbilliviruses and their V proteins in terms of their effects on type I and type II IFN action. These viruses cause disease in different host species, and our results must be viewed in the light that there may be some variation between the viruses' ability to bind to host proteins from different species. However, the host proteins involved in these interactions (STAT1/2, Jak1, Tyk2) are all highly conserved across many species, and we have previously shown that RPV (which causes disease only in cattle, buffalo and related artiodactylates) is highly effective at blocking IFN action in human and monkey as well as bovine cells [23]. We have carried out the studies here in primate cells, choosing Vero cells for transfection and some other studies because they are known not to produce their own type 1 IFN, and support the replication of all morbilliviruses, and A549 cells because they have good levels of endogenous STAT1 and STAT2, making immunofluorescence visualisation of STAT activation better than in the Vero cells. Although we used only one wild type strain of each virus, the activities of these viruses and their proteins in a range of assays has shown that there are several distinct mechanisms employed by the members of this genus to block IFN action, and that they act in synergy to provide maximal ability of the virus to replicate in the face of host innate defences. We observed that V proteins from all four viruses were effective blockers of type I IFN-induced gene transcription using a reporter gene assay, although this was not true of V proteins from two cell culture-attenuated vaccine strains, MeV-Edm and CDV-Ond. However, there were striking differences in the abilities of the V proteins to block type II IFN-induced gene transcription. RPV V was the most effective, and MeV V and CDV V the least, with PPRV V being intermediate. Again the MeV and CDV vaccine strain V proteins were worse than those from their respective unattenuated strains, while there was no significant difference between the V proteins of virulent and vaccine strains of RPV. Similarly, while all the wild-type V proteins were effective in blocking type I IFN-induced STAT1/2 phosphorylation, there was a big variation in their ability to block type II IFN. These data strongly suggested that the mechanism(s) by which the viruses block type I and type II IFN signalling are different.

Studies using a functional assay, the type I IFN-induced establishment of the antiviral state, showed generally similar observations to the other assays of type I IFN signalling, in that the V proteins from all four wild-type morbilliviruses blocked IFNα action, although here we saw differences in the efficacy of the different V proteins, all of which were equally effective in the reporter gene assay and in their ability to block type I IFN-induced STAT1/2 phosphorylation, and this assay may be helpful in unpicking more specific differences between the activity of proteins from different viruses.

Co-precipitation of native STAT1 and/or STAT2 with the paramyxovirus V proteins has been taken by a number of groups as a measure of direct binding of STATs to the V proteins, since STAT1 and STAT2 can only form heterodimers after phosphorylation [16], [18], [26], [32], [33], [34]; however, it has to be born in mind that we could still be looking at the precipitation of another, as yet uncharacterised, complex of V with other host cell proteins, although the variation in the ratio of STAT1 to STAT2 in the V-precipitated complexes argues against this. We found that the different morbillivirus V proteins had different abilities to co-precipitate STAT1 and STAT2. Interestingly, there was a correlation between the ability of the V proteins to bring down STAT1 and their ability to block type II IFN-induced gene transcription or STAT1 phosphorylation. However, there was no correlation between the ability of the V proteins to co-precipitate STAT1 and/or STAT2 and their abilities to block type I IFN-induced gene transcription, phosphorylation of STAT1/STAT2, or the induction of the antiviral state. We therefore looked for another mechanism, apart from STAT binding, that could mediate an effective blockade of type I IFN action. Studies with several other RNA viruses, such as Sendai virus [50], Marbug virus [35], Japanese encephalitis virus [51], [52], Langat virus [53], Dengue virus [54] and West Nile virus [55] have shown interference with the phosphorylation of receptor associated Janus kinases. We found that the virulent strain of RPV (RPV-Sa) had the ability to block type I IFN signalling at this early step in the pathway, blocking the phosphorylation of IFN receptor-associated kinases Jak1 and Tyk2. Since the morbillivirus V proteins, expressed alone, were able to effectively prevent IFNα-induced STAT phosphorylation, we analyzed the ability of the different morbillivirus V proteins to block Jak1/Tyk2 phosphorylation. We found that all the morbillivirus V proteins, but not that of PIV5, could block Tyk2 phosphorylation; however, only the V protein from the virulent strain of RPV (RPV-Sa V) could block Jak1 phosphorylation.

These findings support a hypothesis explaining the differing abilities of the different morbillivirus V proteins to block the type I and type II IFN action. The general ability of the morbillivirus V proteins to block the activity/phosphorylation of Tyk2 enables these proteins to mediate an effective blockade on the type I IFN signalling pathway and hence block the type I IFN-induced antiviral state, irrespective of their ability to bind to STAT1 or STAT2. On the other hand the varied ability of the morbillivirus V proteins to block of type II IFN signalling pathway arises from their differing affinity for STAT1, coupled with the ability of the RPV V protein to block Jak1 activation/phosphorylation. Morbillivirus V proteins that are unable to block Jak1 phosphorylation could still bind STAT1 and at least partially inhibit the type II IFN-induced phosphorylation of STAT1 and activation of gene transcription, while the strong inhibition of IFNγ action by RPV/RPV V protein is explained by its double effect, binding STAT1 and also inhibiting Jak1 activation. The fact that only the RPV-Sa V protein blocked Jak1 phosphorylation is probably not an absolute difference between viruses, but may reflect the specific strains of MeV, CDV and PPRV we used in these studies. Previous work with MeV has shown that infection with this virus can lead to a block in IFNα-induced Jak1 phosphorylation but not Tyk2 phosphorylation [24], while another study with the V protein from a different MeV showed a block in Tyk2 phosphorylation by binding to Jak1 through the P/V/W common domain [15].

We found by co-precipitation studies that the morbillivirus V proteins all interacted with both Jak1/Tyk2, suggesting that either the viral proteins are binding to some common motif on the Janus kinases, or that they are forming a complex with the kinases, possibly at the IFN receptor, and this leads to interference with the activation of the receptor associated kinases. The relatively small amount of the total FLAG-Jak1 or FLAG-Tyk2 that was co-precipitated with the V protein supports the involvement of, and therefore a requirement for, other host cell proteins in a complex with the morbillivirus V. In fact, studies by Yokota et al., 2003 have already proposed a similar mechanism when they observed MeV V and C proteins being associated with IFNAR1 (type I IFN receptor chain 1) and RACK1 (a scaffold protein that links type I IFN receptor chain 2 and STAT1).

Our observations using the VSV-infection assay of the anti-viral state suggest that there is significant overcapacity in the IFN action pathway, and so even a partial transmission of the signal is enough for the cell to be put into the resistant state. The ability of a morbillivirus V protein to interfere at a very early stage in the pathway, with the phosphorylation of Jak1/Tyk2, might be the key factor in enabling these proteins to mediate an effective blockade on type I and/or type II IFN action *in vivo*. Of the viruses in our study, only the Saudi strain of RPV is extremely pathogenic; the RPV-Sa strain is our challenge strain and is 100% fatal in cattle in 7–8 days [56]; the 5804p strain of CDV is fatal in ferrets when administered at a relatively high dose, but still takes 13–14 days [28]. Experimental studies have shown that highly pathogenic RPV replicates more rapidly, resulting in faster and wider *in vivo* distribution [57], [58]. This rapid pathogenesis may be related to the ability of this strain to shut off both type I and type II IFN actions so effectively. It would be interesting to study if there is any correlation between the ability of the morbillivirus V protein to block type II IFN action and the pathogenicity associated with infection.

## Materials and Methods

### Cells and viruses

A549 cells were maintained in Dulbecco's modified Eagle's medium containing 25 mM HEPES buffer, penicillin (100 U/ml), and streptomycin (100 µg/ml) (DMEM) with 5% foetal calf serum. A549-derived stable cell lines were maintained in the same medium with the addition of 2 µg/ml puromycin (GIBCO-Invitrogen) in every passage. Vero cells expressing the human form of the morbillivirus receptor Signalling Lymphocytic Activation Molecule (SLAM) (Vero human-SLAM) (the kind gift of Dr Rick DeSwart, Erasmus Medical College, The Netherlands) were maintained in DMEM containing 5% FCS with the addition of 500 µg/ml G418 (GIBCO-Invitrogen) in every 3rd passage. Vero cells expressing canine SLAM (Vero dog-SLAM) [59] were obtained from Dr. P. Duprex, Queens University of Belfast, UK and were maintained in DMEM containing 10% FCS with the addition of Zeocin (100 µg/ml) (GIBCO-Invitrogen) in every passage. HEK 293FT cells were maintained in DMEM containing 10% FCS. Morbilliviruses used in this study were: RPV-Sa, a virulent strain of RPV isolated from a sample of spleen from a RPV-infected animal [27]; MeV-Dublin, a virulent strain of MeV (the kind gift of Dr. P. Duprex, Queens University of Belfast, UK) [27]; PPRV-Tu 2000, a virulent strain of PPRV [60]; CDV-5804p, a virulent strain of CDV (the kind gift of Dr. P. Duprex, Queens University of Belfast, UK). RPV, MeV and PPRV were grown in Vero human-SLAM cells, and the CDV was grown in Vero dog-SLAM cells. The Indiana strain of vesicular stomatitis virus (VSV) (the kind gift from Dr. N. Ferris, Institute for Animal Health, Pirbright, UK) was grown in Vero cells. RPV and MeV stocks were titred in Vero human-SLAM cells, while PPRV and CDV stocks were titred in Vero dog-SLAM cells, and VSV in A549 cells.

### Plasmids

Plasmids were cloned and grown in *Escherichia coli* (DH5α strain) and purified on CsCl gradients. The pcDNA constructs containing the RPV-Sa P and V open reading frames have been previously described [23], [61]. We constructed pcDNA based expression plasmids for the RPV-Sa W and different morbillivirus V proteins (MeV-Dublin V, MeV-Edm V, PPRV-Tu V, CDV-5804p V and CDV-Ond V) by using PCR and PCR-overlap mutagenesis. For all P, W and V expression constructs, modifications were made to introduce three stop codons into the C open reading frame (ORF) just after the C protein start codon, without altering the P/W/V ORFs and a V5 epitope tag was added to either the 5′ or 3′ end of the coding sequence. Where pre-existing P gene clones or cDNA derived from viral RNA were used as the PCR template, an extra G base was inserted by overlap PCR mutagenesis at the editing site to create a V-type sequence. pcDNA-MeV-Dublin V was generated from pEMC-V^Dub^ (the kind gift of Dr. Linda Rennick, The Queen's University of Belfast, UK) and cloned into the pcDNA vector with a 5′ V5 epitope tag. pcDNA-MeV-Edm V was derived from pMV-Edm-P (plasmid encoding the P protein of MeV Edmonston, the kind gift of Dr. M. Billiter, University of Zurich, Switzerland) and cloned into pcDNA with a 5′ V5 epitope tag. pcDNA-PPRV-Tu-V was generated in a similar fashion from pPPRV-P (plasmid encoding the P protein of PPRV-Tu 2000 [62], and cloned into pcDNA with a 3′ V5 epitope tag. pcDNA-CDV5804p-V was generated from pCDVp5804 (full length plasmid cDNA copy of CDV5804p strain, the kind gift of Dr. Linda Rennick, Queens University of Belfast, UK) and cloned into pcDNA vector with a 5′ V5 epitope tag. pcDNA-CDV-Ond-V was from pCDV-Ond-P (plasmid encoding the P protein of CDV Onderstepoort, the kind gift of Dr. P. Duprex, Queens University of Belfast, UK) and cloned into pcDNA with a 5′ V5 epitope tag. An alignment of the sequences of all the V proteins used in these studies is included as Figure S1.

pcDNA-FLAG-Jak1 was generated by amplifying the Jak1 ORF from cDNA prepared from total A549 RNA and cloned into pcDNA with a 5′ FLAG epitope tag, while pcDNA-FLAG-Tyk2 was similarly generated by amplifying the Tyk2 ORF from pOTB7 (plasmid with human Tyk2 ORF, obtained from the IMAGE clone collection). Plasmids pJAT-lacZ and pGAS-luc were the kind gifts of Prof S. Goodbourn, St. George's Hospital Medical School, London, UK. pGL3-MX1P-luc was the kind gift from Prof. Georg Kochs, Department of Virology, University of Freiburg, Germany. The plasmid encoding the PIV5-V protein (pEF-SV5 V) was the kind gift of Prof R. E. Randal, University of St Andrews, UK. pcDNA-Ganjam virus-N was generated in our laboratory (Holzer et al, 2011). The plasmid encoding the version of RPV-RBOK V with a 5′ V5 epitope tag was generated by PCR from the original clone containing the cMyc-tagged RPV-RBOK V [23]. All PCRs were performed using a proofreading polymerase (KOD; Novagen) and the PCR products introduced into plasmids were sequenced entirely.

### Interferon stocks and antibodies

Recombinant human αA-Interferon was purchased from Calbiochem and human IFNγ was purchased from Millipore. Sources of antibodies were: mouse anti-V5 (AbD Serotech), mouse anti-FLAG (M2 clone, Sigma), mouse anti-STAT1P (pY-701; BD Biosciences), rabbit anti-STAT1 (Upstate, Lake Placid, USA), rabbit anti-STAT2 (Upstate, Lake Placid, USA), rabbit anti-Tyk2P (pY-1054/1055; Cell signalling Technology), rabbit anti-Jak1P (pY-1022/1023; Biosource International, Inc. USA), rabbit anti-Tyk2 (Upstate, Lake Placid, USA), rabbit anti-Jak1 (Upstate, Lake Placid, USA) and mouse anti-PCNA (Santa Cruz Biotechnology). Rabbit anti-RPV (rabbit hyper immune serum raised against RPV) and goat anti-PPRV polyclonal serum were obtained from the livestock morbillivirus reference laboratory, Institute for Animal Health, Pirbright, UK. AlexaFluor 488 or 568-coupled anti-mouse IgG, IgG1, IgG2a or anti-rabbit IgG antibodies were from Invitrogen and the HRP-coupled anti-mouse or anti-rabbit antibodies were from GE Healthcare Life Sciences.

### Transfections and luciferase reporter assays

All transfections were carried out with TransIT LT1 (Mirus) according to the manufacturer's instructions, using a ratio of 3 µl or 2 µl TransIT LT1 per µg DNA. For luciferase reporter assays, Vero human-SLAM cells (10^5^ cells per well) grown overnight in 12 well plates were transfected with a combination of three plasmids (a) 0.5 µg of plasmid of interest (b) 0.5 µg of pJATLacZ and (c) either 0.5 µg of pGL3-MX1P-luc (for the IFNα reporter assays) or 0.5 µg of pGAS-luc (for the IFNγ reporter assays). 24 hours post-transfection, cells were treated with either 1000 IU/ml of IFNα for 8 hours or 1000 IU/ml of IFNγ for 6 hours, or left untreated. The cells were washed once with PBS and then lysed in 200 µl of lysis buffer (50 mM Tris/Cl (pH 7.5), 150 mM NaCl, 2 mM EDTA, 1% (v/v) Nonidet P-40). The samples were centrifuged at high speed for 1 min and 50 µl of the supernatants were mixed with an equal amount of luciferase assay reagent (Promega) and assayed for luciferase activity using an automated Synergy™ 2 multi-detection microplate reader (Biotek). The same samples were then mixed with 150 µl of LacZ assay buffer (48 mM Na_2_HPO_4_, 32 mM NaH_2_PO_4_, 8 mM KCl, 0.8 mM MgSO_4_, 3.2 mg/ml o-nitrophenyl β-D-galactopyranoside) and incubated at 37°C for 30 minutes to measure the β-galactosidase activity (absorbance at 420 nm). The ratio of these two activities was taken as the relative luciferase units (RLUs). Experimental were normalised and statistically analysed as previously described [61]. Multiple comparisons were carried out using Tukey analysis (Minitab) with a maximum probability for significance of p = 0.05.

### Generation of stable A549 cell lines expressing recombinant proteins

Stable cell lines expressing viral proteins of interest, were prepared using the pseudo-typed lentivirus-based vector system described in [63], [64]. The ORFs coding for different proteins were amplified by PCR and cloned into the lentiviral vector pdlNotInPkMCSR, which introduces a 5′ V5 epitope tag. HEK 293FT cells (grown to approximately 50% confluence in 25 cm^2^ flasks) were transfected with 4.5 µg of expression plasmid along with 3 µg of each of the two helper plasmids encoding the VSV G protein (pMD-G) and the Gag/Pol, Tat and Rev proteins (pCMVR8.91) of human immunodeficiency virus type-1 (HIV-1). 48 hours post-transfection, recombinant pseudotyped replication-defective lentiviruses were harvested from the supernatants of the transfected cells. Naive A549 cells (grown to approximately 40% confluence in 25 cm^2^ flasks) were then transduced with the appropriate lentiviral stocks. Puromycin selection was applied at a concentration of 2 µg/ml 48 hours post-transduction to kill un-transduced A549 cells. The population of surviving cells was expanded and grown under puromycin selection. Successful expression of recombinant protein of interest was determined by immunofluorescence and Western blot analysis using mouse anti-V5 antibody.

### VSV cytopathic effect reduction assays

The ability of the viral proteins to overcome antiviral response to IFNα was measured using a cytopathic effect protection assay with VSV as a reporter virus. Briefly, A549-derived stable cell lines were plated in 24-well plates. Next day, the cells were mock treated or treated with 10, 100, 1000 IU/ml of IFNα for 24 hours. The cells were then infected with VSV at a MOI of 0.1 or left untreated. 24 hours post-infection, the cells were fixed using 10% formalin (3.7% formaldehyde solution in PBS) and stained with 0.5% crystal violet.

### Immunofluorescence

For studies on infected cells, A549 cells (1.2×10^5^) grown on 18-mm-diameter cover slips in 12 well plates were either mock infected or infected with RPV-Sa at a nominal MOI of 0.1, with MeV-Dublin or PPRV-Tu at a nominal MOI of 0.5, or with CDV-5804p at a nominal MOI of 1. Eighteen hours post-infection, the cells were treated with either 1000 IU/ml of IFNα or 1000 IU/ml of IFNγ for 30 min or mock treated. The cells were then fixed with 3% paraformaldehyde followed by treatment for 5 mins with cold methanol and blocked with 10% normal goat serum before staining with the appropriate primary and secondary antibodies. To detect infected cells we made use of the strong cross-reaction against all morbilliviruses of polyclonal antisera raised against any one member of the genus. For studies of STAT1 phosphorylation, rabbit hyperimmune serum against RPV (RHIS) was used to detect all viruses. However, for analyzing the activation of STAT2, rabbit anti-STAT2 was used and the accumulation of STAT2 in the nucleus was considered as the marker of phosphorylation/activation of STAT2, as we were unable to find an antibody that could reliably detect phospho-STAT2 in A549 cells. In this case, virus-infected cells were identified using goat anti-PPRV serum which, like the RHIS, cross-reacted with all four morbilliviruses. AlexaFluor-labelled anti-goat and anti-rabbit secondary antibodies were used as required. The nuclei were counterstained with DAPI.

For transfection-based immunofluorescence experiments, A549 cells were transfected with 1 µg of plasmid of interest. 20 hours post-transfection, cells were stimulated with the appropriate IFN and processed as described above. For studies of STAT1 phosphorylation, a combination of mouse anti-STAT1P (IgG1) and mouse anti-V5 (IgG2a) antibodies along with the appropriate isotype-specific AlexaFluor secondary antibodies were used. For studies based on the nuclear translocation of STAT2, a combination of rabbit anti-STAT2 and mouse anti-V5 antibodies were used. Immunofluorescence images were taken on a Leica confocal microscope using sequential scanning at each wavelength and were resized and overlayed using Adobe Photoshop. Confocal microscopy images shown are representative of at least three independent experiments.

### Jak1/Tyk2 over-expression assays

Different amounts of Jak1 and Tyk2 expression plasmids in different cell lines were assessed for the amount of plasmid that gave clear autophosphorylation and detectable FLAG-tagged protein. The amount of viral protein expression plasmid was adjusted to give similar levels of expression indifferent cell lines. For Jak1 experiments, 300 ng of FLAG-Jak1 plasmid was co-transfected with 1000 ng of the plasmid of interest into Vero human-SLAM, while for Tyk2 experiments, 400 ng of FLAG-Tyk2 plasmid was co-transfected with 500 ng of the plasmid of interest into Vero dog-SLAM cells, since phospho-Tyk2 could not be detected in the Vero human-SLAM cell line, presumably due to a high level of PTP1B, the specific phosphatase acting on Tyk2. Twenty-four hours post-transfection, the cells were lysed in SDS-sample buffer containing protease inhibitors (1/200 dilution of protease inhibitor cocktail set III; Calbiochem) and phosphatase inhibitors (50 mM sodium fluoride plus 2 mM sodium orthovanadate; Sigma) and the protein samples were separated on 8% SDS-PAGE gels and immunoblotted with phospho-Jak1 and phospho-Tyk2 antibodies for analyzing the levels of Jak1P and Tyk2P respectively. The levels of FLAG-tagged Jak1 or FLAG-tagged Tyk2 expression in the same samples were analyzed by probing with mouse anti-FLAG antibody, while the expression of viral proteins was assessed using mouse anti-V5.

### Co-immunoprecipitation studies

For STAT1 and STAT2 co-immunoprecipitation experiments, Vero human-SLAM cells (2×10^5^) were grown in 6 well plates and transfected with 1 µg of the plasmid of interest encoding the viral protein. For Jak1 or Tyk2 co-immunoprecipitation experiments, Vero human-SLAM cells (2×10^5^) or 293FT cells (9×10^5^) were grown in 6 well plates and transfected with 1 µg of the plasmid of interest with 2 µg of FLAG-Jak1 or FLAG-Tyk2 plasmid. Forty-eight hours post-transfection, the cells were washed twice with ice-cold PBS and lysed with 500 µl of lysis buffer (50 mM Tris/Cl (pH 7.5), 150 mM NaCl, 2 mM EDTA, 1% (v/v) Nonidet P-40) containing protease inhibitor cocktail set III (Calbiochem) at a final dilution of 1/200. Co-immunoprecipitation experiments requiring lysis buffer at pH 8 were carried out by the same method and with a similar buffer composition, except that the buffer pH was 8. Protein complexes were harvested by immunoprecipitation as previously described [65]. The protein samples were analysed by SDS-PAGE and immunoblotting with specific antibodies. Western blot imaging was carried out with a KODAK 4000R digital imaging system and relative quantitation based on the photon count in the respective bands in the blot, after adjusting for background; comparisons were only made between blots carried out at the same time.

## Supporting Information

Figure S1 **Amino acid sequence alignment of morbillivirus V proteins included in this study.** Amino acid sequences of the V proteins of RPV-Sa, RPV-RBOK, MeV-Du, MeV-Edm, PPRV-Tu, CDV-5804p and CDV-Ond were are shown with shading of conserved residues using GeneDoc. Amino acids highlighted in black are conserved in all the seven different morbillivirus V proteins, while amino acids highlighted in grey are conservative changes, or resides conserved across at least 5 out of 7 sequences.
(TIF)

Table S1 **Comparative abilities of different morbilliviruses and their proteins to block IFN-induced activation of STAT1 and STAT2.**
(DOC)
