# Supplementary figures and images for: Morbillivirus V Proteins Exhibit Multiple Mechanisms to Block Type 1 and Type 2 Interferon Signalling Pathways
Source: PLoS One. 2013 Feb 19;8(2):e57063. doi: 10.1371/journal.pone.0057063 (PMC3576338; doi:10.1371/journal.pone.0057063)

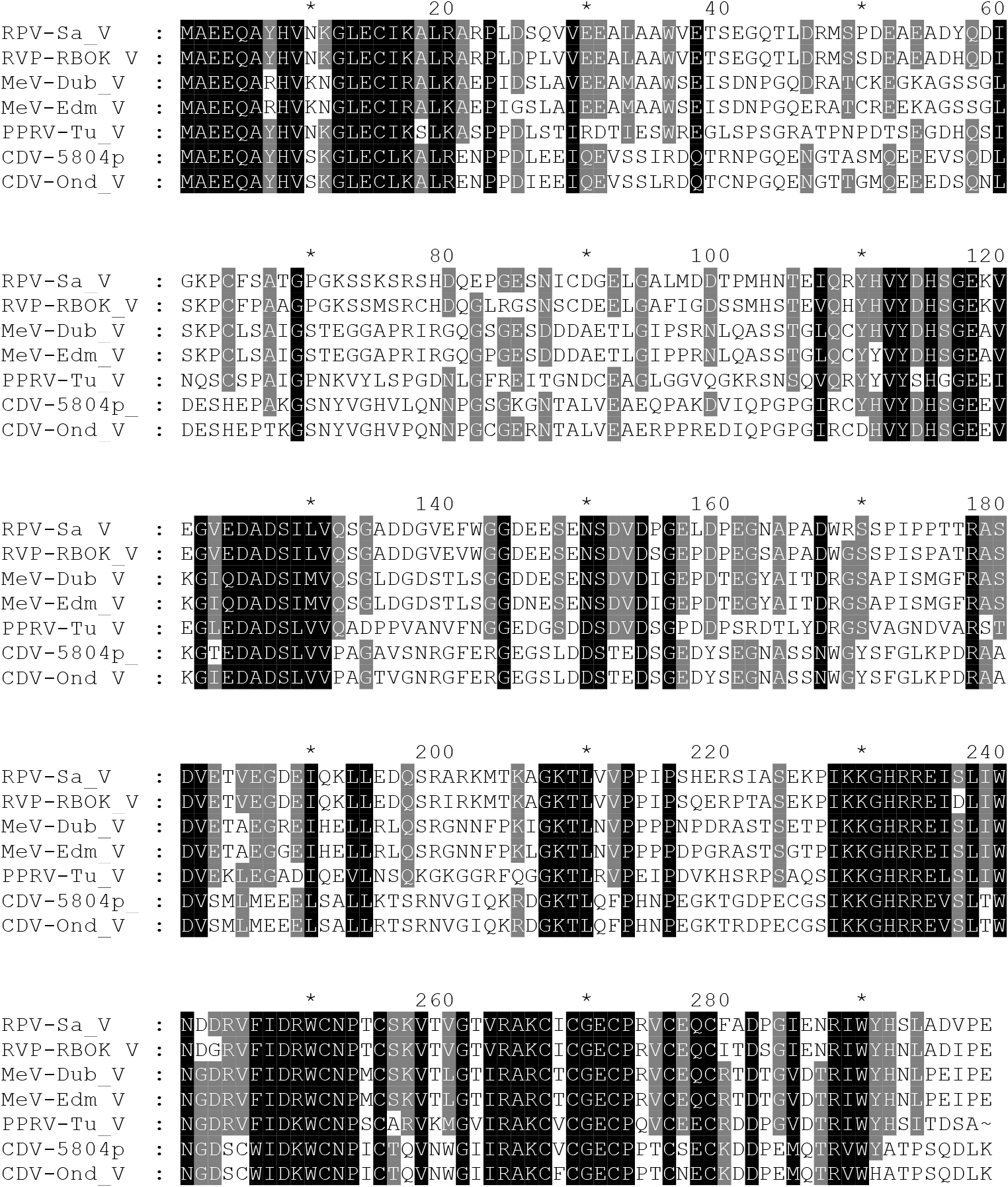

Supplement: Figure S1 — Amino acid sequence alignment of morbillivirus V proteins included in this study. Amino acid sequences of the V proteins of RPV-Sa, RPV-RBOK, MeV-Du, MeV-Edm, PPRV-Tu, CDV-5804p and CDV-Ond were are shown with shading of conserved residues using GeneDoc. Amino acids highlighted in black are conserved in all the seven different morbillivirus V proteins, while amino acids highlighted in grey are conservative changes, or resides conserved across at least 5 out of 7 sequences. (TIF) [file pone.0057063.s001.tif]
