# Supplementary material for: Morbillivirus V Proteins Exhibit Multiple Mechanisms to Block Type 1 and Type 2 Interferon Signalling Pathways
Source: PLoS One. 2013 Feb 19;8(2):e57063. doi: 10.1371/journal.pone.0057063 (PMC3576338; doi:10.1371/journal.pone.0057063)
Supplement: Table S1 — Comparative abilities of different morbilliviruses and their proteins to block IFN-induced activation of STAT1 and STAT2. (DOC) [file pone.0057063.s002.doc]

**Table S1.** Comparative abilities of different morbilliviruses and their proteins to block IFN-induced activation of STAT1 and STAT2.

|  | **IFNα** | | **IFNγ** |
| --- | --- | --- | --- |
| **Virus/Expressed Protein** | **STAT1** | **STAT2** | **STAT1** |
| RPV-Sa | 95 | 100 | 90 |
| MeV-Dub | 61 | 100 | 16 |
| PPRV-Tu | 95 | 100 | 55 |
| CDV-5804p | 57 | 100 | 11 |
| RPV-Sa V | 100 | 100 | 100 |
| RPV-Sa P | 71 | 38 | 69 |
| RPV-Sa W | 64 | 32 | 66 |
| GFP | 22 | 18 | 20 |
| GFP-Vs | 45 | 34 | 42 |
| RPV-RBOK V | 100 | 94 | 100 |
| MeV-Dub V | 87 | 94 | 51 |
| MeV-Edm V | 43 | 18 | 37 |
| PPRV-Tu V | 94 | 94 | 72 |
| CDV-5804p V | 90 | 93 | 53 |
| CDV-Ond V | 55 | 48 | 40 |

The table shows the percentage of cells infected with different morbilliviruses, or expressing detectable amounts of the viral protein, in which activation of STAT1 (defined as appearance of phosphorylated STAT1 in the nucleus) or activation of STAT2 (defined as translocation of additional STAT2 to the nucleus) in response to IFNα or IFNγ was blocked.
